# T-stage-specific abdominal visceral fat, haematological nutrition indicators and inflammation as prognostic factors in patients with clear renal cell carcinoma

**DOI:** 10.1080/21623945.2022.2048546

**Published:** 2022-03-12

**Authors:** Hao Guo, Yumei Zhang, Heng Ma, Peiyou Gong, Yinghong Shi, Wenlei Zhao, Aijie Wang, Ming Liu, Zehua Sun, Fang Wang, Qing Wang, Xinru Ba

**Affiliations:** a Department of Radiology, Qilu Hospital, Cheeloo College of Medicine, Shandong University, Shandong province, Jinan, China; b Department of Radiology, Yantai Yuhuangding Hospital, Shandong province, Yantai, China; c Department of Radiology, Lanshan Branch of Yantai Yuhuangding Hospital, Shandong province, Yantai, China; d Department of Radiology, Yaitai Shan Hospital, Shandong province, Yantai, China

**Keywords:** Obesity, clear renal cell carcinoma, T-stage, abdominal, computed tomography, prognosis

## Abstract

Clear cell renal carcinoma (ccRCC) is the most common histological type of renal cancer and has the highest mortality. Several studies have been conducted on the relationship between adipose tissue and ccRCC prognosis, however, the results have been inconsistent to date. The current study aimed at establishing a link between abdominal fat composition and short-term prognosis in patients with ccRCC after T-stage stratification. We retrospectively analysed 250 patients with pathologically confirmed ccRCC (173 low T-stage and 77 high T-stage) in our hospital. The computed tomography (CT) images were evaluated using ImageJ. Then, subcutaneous and visceral fat areas (SFA and VFA), total fat areas (TFA) and the relative VFA (rVFA) were measured and computed. Meanwhile, biochemical indices of blood serum were analysed. The results showed that rVFA in low T-stage cohort who had a history of short-term postoperative complications were significantly lower than those who did not. No such association was observed in the high T-stage cohort. Further investigation revealed that the correlations between biochemical indexes and fat area-related variables varied across T-stage groups. As a result, rVFA is a reliable independent predictor of short-term prognosis in patients with low T-stage ccRCC but not in patients with high T-stage ccRCC.

## Introduction

Renal cell carcinoma (RCC) is a major cause of cancer-related mortalities [1], accounting for 4% of all new cancer cases worldwide [2]. Like most common tumours, RCC progression is often associated with nutritional and functional changes [3,4]. Generally, a sharp increase in the overweight and obese population in recent years has accelerated obesity-related health problems [5]. For example, Gaetano et al. [6] demonstrated that obesity affects the progressive disease course in RCC. Although the precise mechanisms underlying the association between obesity and tumour progression remain unclear, researchers have suggested multiple possible pathways linking adipose tissue to cancer. These include modification of the immediate surrounding hormone micro-environment of the adipose tissue, chronic inflammatory response, chronic tissue hypoxia and dysfunction of metabolism and/or immunity [7,8]. Previous studies have mainly adopted weight and body mass index (BMI) to define obesity [5,9]. However, these variables do not effectively discriminate between adipose and lean mass distribution, and have also been associated with failure to capture its underlying biology [10,11]. At present, computed tomography (CT) is the gold-standard technique for assessing body composition [12]. Numerous studies have shown the effectiveness of CT-based measures of fat distribution in predicting tumour progression [13,14,15]. For example, results from a CT-based assessment of 1473 patients with gastrointestinal cancer and 273 patients with metastatic RCC revealed that subcutaneous adipose tissue index (SATI) was positively associated with survival rates of patients [16]. Meanwhile, findings from another study comprising 114 patients with metastatic RCC revealed that visceral fat accumulation was correlated with both longer progression-free and overall survival (PFS and OS) [17]. Recently, visceral fat was found to be positively correlated with survival periods of patients with advanced RCC, but negatively associated with those of patients with liver cancer [18,19]. This contradiction underlines the complexity of obesity-related outcomes. The possible explanation for this phenomenon is partly attributed to inconsistencies in the cancer subtypes and stage of samples used or imperfect treatments. Therefore, we propose that the power of obesity association studies may increase when disease heterogeneity, such as tumour type, and stage, among others, be taken into account. The aim of the present observational study was to explore the relationship between adipose tissue and short-term prognosis of ccRCC patients, targeting the T-stage factors. Furthermore, we incorporated haematological indicators related to nutrition and inflammation to investigate the possible mechanism of action.

## Materials and methods

### Patients recruitment and selection criteria

Data analysed in this study were retrieved from the Picture Archiving and Communication System (PACS) database of Yantai Yuhuangding hospital (China), between January 2014 and April 2021. The retrospective study was approved by the hospital’s ethics review committee, who also waived informed consent. Patients were recruited in the study if they exhibited definite pathological T-stage of ccRCC. Conversely, those with other renal tumours, systemic metabolic disorders, more than 3% variation in body weight in the previous three months as well as those with unavailable preoperative CT data, were excluded. Data were performed with all clinical and pathological information hidden. Data for 250 patients (176 males and 74 females) were retrospectively collected, 30 days after surgery. Short-term complications included surgical site infection/haemorrhage, bowel dysfunction, urinary tract infection, fever, respiratory failure, acute kidney injury and lower limb venous thrombosis. On the other hand, clinical information included patient’s age, gender, surgical procedures, complications, T-stage and biochemical data. All patients were divided into low T-stage (T1 and T2) and high T-stage (T3 and T4) groups, as previously described [20].

### Analysis of fat composition

**Figure.1.**
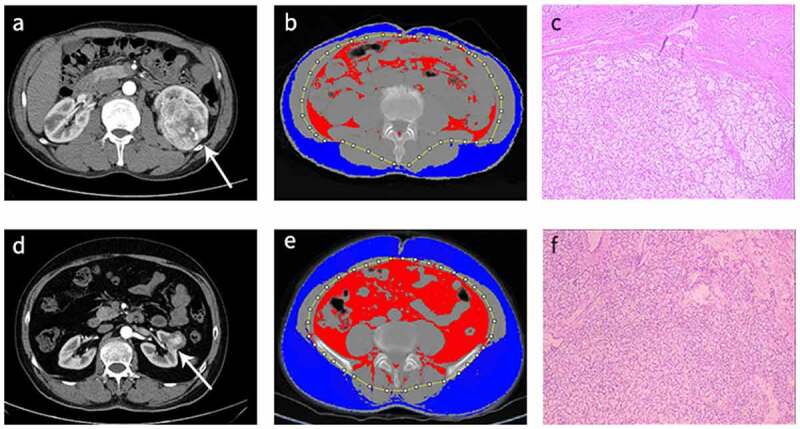
Representative preoperative images of ccRCC patients in low T-stage. (a-c) Low T-stage ccRCC patient with short-term postoperative complications in a 50-year-old man. (d-f) Low T-stage ccRCC patient without short-term postoperative complications in a 58-year-old man. (a, d) Contrast-enhanced axial CT scan images showing tumors in the left renal (white arrow). (b, e) CT images (blue areas represented regions which are subcutaneous fat, and red areas represent regions which are visceral fat) with relative visceral fat area (rVFA) of 30.2% (b) and 39.0% (e). (c, f) Pathology of the tumors showing low T-stage ccRCC.

CT images were extracted from PACS and imported into ImageJ 1.51 [18,21,22]. Preoperative abdominal CT axial plain scan images were used to perform the analyses at the level of the umbilical as previously described [18,^21–23^]. Region of interest measurements were divided into subcutaneous fat area (SFA) and visceral fat area (VFA), based on inside and outside the abdominal wall using standard Hounsfield unit (HU) threshold ranges (−150 to −50) (Figure 1). Total fat area (TFA) was calculated using the formula: TFA = VFA + SFA, whereas relative VFA (rVFA) was calculated as follows: rVFA = (VFA/TFA) × 100%. All measurements were performed by two independent experienced senior radiologists, who were blinded to patients’ clinical information.

### Statistical analysis

Continuous variables, which conformed to normal distribution and homogeneity of variance, were presented as means ± standard deviations (SD), and were subjected to a *t*-test to assess differences between groups. Non-normally distributed data were presented as medians (quartiles) and subjected to the Wilcoxon rank-sum test to determine differences between groups. Categorical variables were presented as frequencies (percentages), and differences among groups compared using the chi-square test. Significant predictors of short-term postoperative complications in patients with ccRCC were first examined using the univariate logistic regression model, followed by a bidirectional stepwise regression analysis. Area under the curve (AUC), of the receiver operating characteristic (ROC) were utilized to assess the predictive performance of significant predictors. Correlations between fat area-related variables and biochemical indicators were performed using Spearman’s and Pearson’s correlation tests. All statistical analyses were carried out using packages implemented in R software v4.0.2, with the two-tailed test (*p* < 0.05) considered statistically significant.

## Results

### Clinical characteristics

A total of 250 ccRCC patients (176 men and 74 women) with a definite pathological diagnosis (173 low T-stage and 77 high T-stage) were enrolled in this study. Baseline clinical characteristics of patients in the two groups are outlined in Table 1. Summarily, patients with short-term postoperative complications were associated with significantly lower VFA (110.0 vs 148.1, *p* < 0.0001) and rVFA (33.6 vs 47.5, *p* < 0.0001), as well as higher SFA (201.9 vs 171.3, *p* = 0.007), relative to those without them in low T-stage group. However, we found no statistically significant differences between fat area-related variables and short-term postoperative complications in the high T-stage group.

**Table 1.** The baseline characteristics, preoperative biochemical indexes and fat measurements of patients with (Yes) and without (No) short-term postoperative complications in low T-stage and high T-stage

	Low T-stage (n = 173)			High T-stage (n = 77)		
Variable	No	Yes	*p*	No	Yes	*p*
Age, year	58.4 ± 9.71	57.4 ± 9.6	0.496^a^	63 (57, 70)	61 (53.5, 66.0)	0.394^c^
Gende, n (%)						
Female	20 (17.7)	34 (56.7)		19 (33.3)	1 (5.0)	
Male	93 (82.3)	26 (43.3)	**<0.0001**^b^	38 (66.7)	19 (95.0)	0.029^b^
Neutrophils, × 10^9^/L)	3.5 (2.8, 4.4)	3.0 (2.5, 3.8)	0.030^c^	3.9 ± 1.2	4.05 ± 1.4	0.649^a^
Lymphocytes, × 10^9^/L	1.7 (1.4, 2.1)	1.5 (1.3, 2.0)	0.117^c^	1.6 (1.4, 2.2)	1.5 (1.1, 1.7)	0.162^c^
Platelet count, × 10^9^/L	225 (196, 266)	227.5 (194.3, 272.3)	0.795^c^	259 (218, 311)	283.5 (203.0, 335.8)	0.719^c^
Serum albumin, g/L	40.9 (38.6, 44.2)	41.2 (39.1, 44.4)	0.700^c^	39.4 (35.4, 42.7)	40.1 (35.9, 43.5)	0.609^c^
HSP90α, pg/ml	64.4 (51.0, 93.6)	66.8 (52.9, 107.4)	0.451^c^	72.7 (39.9, 92.1)	67.4 (58.0, 96.0)	0.524^c^
NLR	2.0 (1.4, 2.8)	2.0 (1.5, 2.7)	0.773^c^	2.1 (1.6, 3.0)	3.1 (2.2, 3.3)	0.080^c^
PLR	131.1 (103.5, 165.1)	138.3 (105.3, 187.3)	0.208^c^	140 (112.2, 186.4)	151.0 (121.0, 227.8)	0.374^c^
SII	428.1 (318.3, 668.7)	416.8 (293.8, 619.7)	0.647^c^	528.6 (367.2, 828.0)	721.6 (422.8, 932.0)	0.338^c^
PNI	50.1 (46.5, 53.3)	49.9 (46.3, 53.9)	0.770^c^	48.7 (43.1, 53.2)	47.8 (41.7, 51.2)	0.732^c^
VFA, cm^2^	148.1 (112.3, 194.0)	110.0 (71.6, 133.5)	**< 0.0001^c^**	147.4 ± 59.1	144.3 ± 62.6	0.845^a^
SFA, cm^2^	171.3 (138.2, 206.9)	201.9 (142.3, 286.6)	**0.007**^c^	180.7 (122.6, 212.7)	150.5 (123.3, 176.2)	0.169^c^
TFA, cm^2^	335.5 (263.3, 390.4)	327.0 (219.7, 414.3)	0.820^c^	335.0 ± 131.6	296.1 ± 114.7	0.218^a^
rVFA, %	47.5 ± 7.2	33.6 ± 8.4	**< 0.0001**^a^	44.7 ± 10.4	47.5 ± 8.3	0.223^a^

*p* < 0.05 is indicated by boldface

Relationship between T-stage-specific fat area-related variables with short-term postoperative complications.

A summary of results on the relationship between T-stage-specific fat area-related variables and with short-term postoperative complications is outlined in Table 2. Results from univariate logistic regression analysis revealed that gender (OR 6.081, 95% CIs 3.049–12.494, *p* < 0.0001), VFA (OR 0.979, 95% CIs 0.971–0.987, *p* < 0.0001), SFA (OR 1.008, 95% CIs 1.003–1.012, *p* = 0.001) and rVFA (OR 0.793, 95% CIs 0.734–0.845, *p* < 0.0001) were significantly associated with short-term postoperative complications in the low T-stage group. In the high T-stage group, only gender (OR 0.105, 95% CIs 0.006–0.569, *p* = 0.034), but not any other fat area-related variables, was a significant independent predictor of patient prognosis. Consequently, all significant variables with prognostic value in univariate logistic regression were incorporated into bidirectional stepwise regression, and their relationship with short-term postoperative complications evaluated. Results revealed that two variables (VFA and rVFA) and one variable (gender) were screened out in the low and high T-stage groups, respectively. Results from multivariate analyses demonstrated that rVFA (OR 0.809, 95% CIs 0.746–0.865, *p* < 0.0001) and gender (OR 0.105, 95% CIs 0.006–0.569, *p* = 0.034) were significant predictors of short-term postoperative complications in the low and high T-stage groups, respectively.

**Table 2.** Univariate and multivariate logistic regression analysis of the relationship between T-stage-specific fat area-related variables and short-term postoperative complications

	Univariate		multivariate		Univariate		multivariate	
Variable	OR (95% CI)	*p*	OR(95% CI)	*p*	OR (95% CI)	*p*	OR(95% CI)	*p*
Age, year	0.989 (0.957–1.022)	0.495			0.991 (0.942–1.046)	0.741		
Gender	6.081 (3.049–12.494)	**<0.0001**			0.105 (0.006–0.569)	**0.034**	0.105(0.006–0.569)	**0.034**
Neutrophils, × 10^9^/L	0.826 (0.638–1.041)	0.123			1.114 (0.726–1.717)	0.618		
Lymphocytes, × 10^9^/L	0.691 (0.393–1.112)	0.170			0.478 (0.169–1.189)	0.133		
Platelet count, × 10^9^/L	1.001 (0.996–1.006)	0.680			0.999 (0.993–1.006)	0.824		
Serum albumin, g/L	0.999 (0.944–1.053)	0.977			1.008 (0.921–1.097)	0.853		
HSP90α, pg/ml	1.004 (0.997–1.011)	0.289			1.004 (0.988–1.020)	0.600		
NLR	0.950 (0.710–1.249)	0.718			1.275 (0.824–1.980)	0.267		
PLR	1.004 (0.999–1.010)	0.139			1.003 (0.996–1.009)	0.384		
SII	1.000 (1.000–1.001)	0.781			1.000 (0.999–1.002)	0.522		
PNI	0.980 (0.930–1.027)	0.422			0.982 (0.910–1.055)	0.633		
VFA, cm2	0.979 (0.971–0.987)	**<0.0001**	0.992(0.982–1.002)	0.148	0.999 (0.990–1.008)	0.837		
SFA, cm2	1.008 (1.003–1.012)	**0.001**			0.995 (0.987–1.001)	0.143		
TFA, cm2	0.999 (0.997–1.002)	0.669			0.998 (0.993–1.002)	0.243		
rVFA, %	0.793 (0.734–0.845)	**<0.0001**	0.809(0.746–0.865)	**<0.0001**	1.031 (0.978–1.089)	0.268		

### Correlation between biochemical blood serum indexes with fat area-related variables

Results from the correlation between biochemical blood serum indexes of the blood serum and with fat area-related variables are outlined in Table 3. Notably, we employed the Shapiro–Wilk test to check data normality, then applied either Pearson’s or Spearman’s correlation coefficients to perform correlations between various analysed parameters, depending on the normality distribution of variables. In low T-stage group, we found positive correlations between heat shock protein (HSP) 90α with TFA (*r* = 0.288, *p* < 0.001) and SFA (*r* = 0.289, *p* < 0.001), neutrophils with VFA (*r* = 0.215, *p* = 0.004) and rVFA (*r* = 0.309, *p* < 0.001), and lymphocyte with VFA (*r* = 0.166, *p* = 0.03) and rVFA (*r* = 0.151, *p* = 0.047). In the high T-stage group, prognostic nutritional index (PNI) was positively correlated with TFA (*r* = 0.294, *p* = 0.01) and SFA (*r* = 0.307, *p* = 0.007), whereas serum albumin was positively associated with TFA (*r* = 0.284, *p* = 0.012) and SFA (*r* = 0.29, *p* = 0.011). In the high T-stage group, negative correlations were detected between systemic immune-inflammation index (SII) with TFA (*r* = −0.256, *p* = 0.025) and SFA (*r* = −0.251, *p* = 0.028), neutrophil-to-lymphocyte ratio (NLR) with SFA (*r* = −0.257, *p* = 0.024), and lymphocytes with rVFA (*r* = −0.24, *p* = 0.036).

**Table 3.** Relationship between preoperative biochemical indexes and fat area-related variables

	TFA		SFA		VFA		rVFA		TFA		SFA		VFA		rVFA	
	Cor	*p*	Cor	*p*	Cor	*p*	Cor	*p*	Cor	*p*	Cor	*p*	Cor	*p*	Cor	*p*
Neutrophils, × 10^9^/L	0.014	0.853^b^	−0.103	0.178^b^	0.215	**0.004**^b^	0.309	**<0.001**^b^	−0.128	0.267^a^	−0.155	0.178^b^	−0.065	0.572^a^	0.066	0.568^a^
Lymphocytes,, × 10^9^/L	0.114	0.134^b^	0.058	0.447^b^	0.166	**0.030**^b^	0.151	**0.047**^b^	0.123	0.286^b^	0.185	0.107^b^	−0.076	0.509^b^	−0.240	**0.036**^b^
Platelet count, × 10^9^/L	0.043	0.576^b^	0.024	0.752^b^	0.049	0.522^b^	0.051	0.506^b^	−0.172	0.136^a^	−0.128	0.269^b^	−0.135	0.243^a^	−0.02	0.861^a^
Serum albumin, g/L	0.016	0.831^b^	0.045	0.559^b^	−0.012	0.875^b^	−0.076	0.319^b^	0.284	**0.012**^b^	0.290	**0.011**^b^	0.187	0.104^b^	−0.038	0.743^b^
HSP90α, pg/ml	0.288	**<0.001**^b^	0.289	**<0.001**^b^	0.166	0.057^b^	−0.106	0.227^b^	−0.055	0.726^b^	−0.089	0.570^b^	0.011	0.943^b^	0.170	0.275^b^
NLR	−0.075	0.325^b^	−0.114	0.136^b^	0.029	0.705^b^	0.101	0.188^b^	−0.216	0.059^b^	−0.257	**0.024**^b^	−0.011	0.921^b^	0.197	0.086^b^
PLR	−0.083	0.280^b^	−0.053	0.491^b^	−0.118	0.122^b^	−0.092	0.229^b^	−0.217	0.058^b^	−0.215	0.060^b^	−0.048	0.675^b^	0.144	0.211^b^
SII	−0.040	0.597^b^	−0.094	0.217^b^	0.062	0.420^b^	0.131	0.085^b^	−0.256	**0.025**^b^	−0.251	**0.028**^b^	−0.100	0.387^b^	0.097	0.400^b^
PNI	0.068	0.378^b^	0.069	0.372^b^	0.069	0.368^b^	0.005	0.945^b^	0.294	**0.010^b^**	0.307	**0.007**^b^	0.143	0.214^b^	−0.108	0.351^b^

### Validation of the predictive function of rVFA

**Figure.2.**
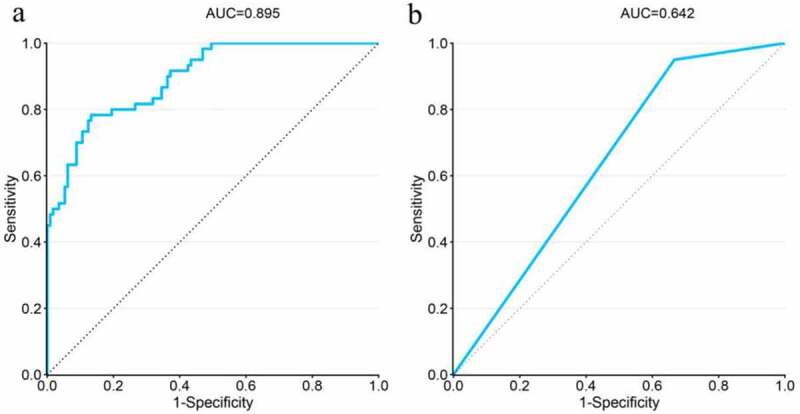
ROC curve of the prediction models. (a) Results showed that rVFA demonstrated highly accuracy for prediction of short-term postoperative complications of low T-stage ccRCC patients. (b) Gender was a weak predictor of short-term postoperative complications of high T-stage ccRCC patients.

rVFA was highly accurate in predicting short-term postoperative complications in patients with low T-stage ccRCC, as evidenced by an AUC value of 0.895. Conversely, gender was a weak predictor of poor outcomes in patients with high T-stage ccRCC (AUC = 0.642) (Figure 2).

## Discussion

In the present study, we examined the short-term prognostic impact of T-stage-specific fat distribution in ccRCC patients. Our results demonstrated that relative loss of visceral fat is associated with a high risk of postoperative complications in ccRCC patients with low T-stage, suggesting that high visceral fat may have a protective role in this group of patients. Relative visceral fat is a reliable biomarker for identifying low T-stage, but not advanced ccRCC patients who may have an inferior short-term prognosis after undergoing laparoscopic nephrectomy.

Although surgical resection is the primary therapy for treating patients with ccRCC, the value of comprehensive treatment, such as chemotherapy and radiotherapy, remains uncertain. Previous studies have shown that any invasive procedure is likely to be accompanied by various complications such as haemorrhage, and infections, as well as severe complications like multiorgan failure or death [24]. Notably, predicting postoperative complications allows clinicians to take personalized treatment and may improve the prognosis. Although previous studies have shown that BMI is an independent predictor of perioperative complications [25], it does not precisely reflect individual differences of fat distribution [26]. Researchers have suggested that fat distribution pattern is a reliable predictor of obesity-related outcomes [27]. Recently, other precision approaches of reflecting fat distribution, namely CT and MRI, have emerged with the aim of ascertaining the relationship between fat composition and onset of complications following surgery [17,18,28,29]. Results from a previous study demonstrated that greater VFA was associated with favourable overall survival of patients with advanced ccRCC, suggesting that visceral fat may be a marker for nutritional status [29]. Additional research evidences found that visceral fat but not BMI, was a positive biomarker for predicting recurrence-free survival in patients with localized ccRCC [18]. Moreover, lower VAT was associated with high risk of overall survival in advanced RCC patients who underwent nephrectomy [22]. This VAT-related survival advantage has also been demonstrated in patients with metastatic RCC [17]. Conversely, some scholars have reported that VFA is negatively correlated with progression of metastatic RCC [30]. Coincidentally, this contradictory phenomenon was not unique. Results from a previous study demonstrated that visceral obesity was associated with longer operating times in laparoscopic radical nephrectomy [21], while Ioffe et al. [31] found that VAT thickness was not associated with operation time, complications and warm ischaemia time among 118 patients who underwent nephrectomy. In addition, some researchers have demonstrated that rVFA was positively correlated with the risk of death in female ccRCC patients [32]. However, results from another study showed that rVFA had no significant influence on survival outcomes in patients with ccRCC [22]. Taken together, these findings affirm the complexity and heterogeneity of the adipose tissue.

Based on this, we stratified patients into different T-stage groups and evaluated the effect of T-stage-specific abdominal visceral fat on short-term prognosis in ccRCC patients. To our knowledge, this is the first report of this phenomenon. Our findings revealed that low rVFA levels were significantly associated with a high risk of short-term complications following either laparoscopic partial or radical nephrectomy in ccRCC patients with low T-stage. This indicates that VFA exerts a post-operative protective effect in ccRCC patients with low T-stage. Our results differ from those of Zhai et al. [33], who found that higher VFA may confer higher risk of postoperative complications in ccRCC patients. However, their study did not consider tumour staging, a phenomenon that might explain the inconsistent results. The observed association between short-term postoperative complications with rVFA in patients with low T-stage may be attributed to several reasons. Firstly, a reduction in adipose tissue might reflect a metabolism alteration, including malnutrition or lipodystrophy. Previous studies have shown that low visceral fat content is an indicator for poor systemic status (pre-cachectic), thus portends a poor response to standard cancer treatments [34–36]. Secondly, adipose tissue plays a crucial role in body energy reserves, while fat mobilization compensates the energy deficits under large negative energy balance in obese patients [37]. In contrast, low visceral fat levels indicate low body energy reserves, which is a predictor for poor patient outcome [18,22]. Recent studies have revealed the mechanism underlying the effect of perirenal fat/ccRCC in tumour progression [38]. However, the mechanism underlying regulation of rVFA expression during tumour progression in cells remains unclear. Notably, some studies have suggested that up-regulation of peroxisome proliferator-activated receptor gamma coactivator-1α (PGC1α) and uncoupled protein 1 (UCP1) expression in adipose tissues leads to a robust resistance to obesity and related diseases [39,40]. Functionally, the adipose tissue secretes various adipokines and cytokines, which can systemically regulate physiological functions through endocrine-like activity. These factors have been implicated in promotion of tumour development and progression [41]. Apart from this mechanism, fatty acids are also important in cell proliferation and tumour progression, where they serve as building blocks for membrane synthesis and act as signalling molecules [42]. Some studies have reported that obese people have a low expression of the fatty acid synthase (FASN) gene [43], which encodes the enzyme fatty acid synthase that is responsible for secretion of fatty acids – an essential source of energy. The altered gene expression may have led to slower-growing kidney tumours. Notably, we found no definite association between postoperative complications with either VFA or SFA in high T-stage group, suggesting that adipose tissue is not a significant prognostic predictor for patients with advanced ccRCC. The choice of treatment and performance status of patients are probably more reflective in prognosis of this group of patients.

Our results further revealed a positive correlation between HSP90α with both SFA and TFA in low T-stage ccRCC patients. Previous studies have shown that stress response can stimulate expression of HSPs, thereby improving stress resistance [44,45]. Notably, high levels of HSP70 in serum have been associated with improved insulin sensitivity and increased adipose tissue [46,47]. For example, Qu et al. [48] found that heat stress (HS) was significantly associated with increased production of adipose tissue in pigs, and this phenomenon was attributed to upregulation of fatty acid transport and glyceroneogenic genes. However, some researchers have reported that HSP70 expression is positively associated with insulin resistance [49]. A recent study found that obesity was associated with upregulation of HSP90 in testicular tissues, but an increase in HSP90 out of proportion to proliferating cell nuclear antigen (PCNA) could not exert its physiological effects, including complete the DNA repair pathways and maintain the cellular DNA integrity [50]. HSP90 expression has also been found to be a potential biomarker for prognosis of patients with some cancers. Lin et al. [51] showed that high expression of HSP90AA1, HSP90AB1 and HSP90B1 was associated with poor prognosis of breast cancer patients. Another study showed that HSP90 was generally overexpressed in malignant paediatric brain cancer, and this expression was correlated with that of HSP70 [52]. Bayer et al. [53] proved that HSP70 was a tumour-specific biomarker that could be used to monitor the effect of radiation therapy in mice tumour models, and suggested that soluble HSP70 was useful in detecting tumours at early stages. Collectively, these findings indicate that HSPs are potential markers and anticancer targets due to their ability to regulate key events during development of malignant tumours. However, the relationship between profiles of HSP expression and their activity with cancer diagnosis, patient prognosis, metabolism and treatment remains unclear. Results of the present study demonstrated that HSP90α upregulation was not directly associated with short-term outcomes and VFA of any T-stage ccRCC patients. This indicates that each functional HSP process does not act independently, but involves some other factors, such as specific tumour types that act at specific stages, and fat distribution, among others, to achieve its biological function. However, further studies are needed to elucidate this complex mechanism.

Adipocytes produce a variety of mediators, including adipokines, inflammatory cytokines and tumour necrosis factors [54]. Previous studies have shown that inflammation, as well as immune and nutritional status are closely related to prognosis of patients with several tumours [55,56]. For example, IL-6 levels were significantly correlated with the risk of first decompensation in patients with advanced chronic liver disease as well as death/transplantation in those with advanced chronic liver disease [57]. On the other hand, Zhang et al. [58] elaborated the prognostic value of serum inflammation biomarkers in early-stage lung adenocarcinoma. Moreover, some scholars have postulated that inflammation is part of the active cross-talk between the tumour and the host, while its microenvironment could be affecting prognosis of patients with gastric carcinoma [59]. Notably, both albumin (Alb) ≥ 40 g/l and lymphocyte-to-monocyte ratio (LMR) ≥ 3.4 were found to be protective factors for gastric cancer (GC) [56], indicating that inflammation is relatively beneficial under certain situations for tumour prognosis. The mechanism underlying this relationship has been found to be both complex and multidirectional. Results of the present study demonstrated that preoperative neutrophil and lymphocyte levels were positively correlated with VFA and rVFA in patients with low T-stage, suggesting that VFA could be associated with systemic inflammation. Results from previous studies have suggested that VFA and SFA not only have different inflammatory properties but also generate different inflammatory responses [60,61].

In the present study, rVFA was strongly correlated with short-term prognosis of ccRCC patients with low T-stage, but we found no significant association between preoperative inflammation indexes with short-term outcomes. We speculated that preoperative inflammation status, tumour stage and VFA may have synergistic effects on short-term prognosis. Furthermore, the correlations between preoperative inflammation indexes, prognostic nutrition index (PNI), HSP90α and adiposity parameters were different in high T-stage groups. Notably, HSP90α was no longer correlated with both TFA and SFA, although lymphocytes were negatively associated with rVFA in ccRCC patients with high T-stage. Similarly, NLR and SFA also exhibited a negative correlation, with a similar pattern observed between systemic immune-inflammation index (SII) and some fat area-related variables (TFA and SFA). Meanwhile, potential nutritional markers, such as PNI and serum albumin, were positively associated with TFA and SFA. We also observed significant differences between preoperative inflammation indexes and adiposity parameters relative to the low T-stage group. This discrepancy may suggest that inflammatory conditions may have a wide variation among different tumour stage, a phenomenon that should be taken into account when designing immunotherapeutic strategies. Interestingly, although nutritional state and fat reserves were strongly correlated in the high T-stage group, neither of them was associated with short-term prognosis. This finding possibly indicates that factors determine the prognosis of high T-stage ccRCC patients may have other priorities such as the presence or absence of metastasis.

This study had some limitations. Firstly, our sample size was relatively small, and only patients with electronically available CT scans were included. Secondly, being a single-centre study there is a possibility of bias. Finally, we did not perform subgroups analyses using the Clavien–Dindo grading system due to the smaller variety of complications in the recruited patients.

In conclusion, our results indicate that visceral fat has a protective effect on short-term postoperative complications among low T-stage ccRCC patients. Moreover, RVFA may be a reliable predictive marker for short-term postoperative complications in early-stage ccRCC patients undergoing laparoscopic nephrectomy. Preoperative inflammation and nutritional indexes, HSP, fat distribution and tumour stage may have synergistic effects on short-term prognosis.

## Data Availability

The authors confirm that the data supporting the findings of this study are available within the article and its supplementary materials (https://doi.org/10.5281/zenodo.6334239).
